# Myofibroblast-Derived SFRP1 as Potential Inhibitor of Colorectal Carcinoma Field Effect

**DOI:** 10.1371/journal.pone.0106143

**Published:** 2014-11-18

**Authors:** Gábor Valcz, Árpád V. Patai, Alexandra Kalmár, Bálint Péterfia, István Fűri, Barnabás Wichmann, Györgyi Műzes, Ferenc Sipos, Tibor Krenács, Emese Mihály, Sándor Spisák, Béla Molnár, Zsolt Tulassay

**Affiliations:** 1 Molecular Medicine Research Unit, Hungarian Academy of Sciences, Budapest, Hungary; 2 2nd Department of Internal Medicine, Semmelweis University, Budapest, Hungary; 3 1st Department of Pathology and Experimental Cancer Research, Semmelweis University, Budapest, Hungary; 4 Dana-Farber Cancer Institute, Harvard Medical School, Boston, Massachusetts, United States of America; Fox Chase Cancer Center, United States of America

## Abstract

Epigenetic changes of stromal-epithelial interactions are of key importance in the regulation of colorectal carcinoma (CRC) cells and morphologically normal, but genetically and epigenetically altered epithelium in normal adjacent tumor (NAT) areas. Here we demonstrated retained protein expression of well-known Wnt inhibitor, secreted frizzled-related protein 1 (*SFRP1*) in stromal myofibroblasts and decreasing epithelial expression from NAT tissues towards the tumor. *SFRP1* was unmethylated in laser microdissected myofibroblasts and partially hypermethylated in epithelial cells in these areas. In contrast, we found epigenetically silenced myofibroblast-derived SFRP1 in CRC stroma. Our results suggest that the myofibroblast-derived SFRP1 protein might be a paracrine inhibitor of epithelial proliferation in NAT areas and loss of this signal may support tumor proliferation in CRC.

## Introduction

Communication between the epithelial and stromal compartments is fundamental in the modulation of normal epithelial homeostasis which becomes dysregulated during carcinogenesis due to genetic and epigenetic alterations [1]. Regarding these compartments myofibroblasts (α-SMA+, fibroblast-like cells) represent ultimate members of the information flow [2]. Myofibroblasts become primarily mesenchymal elements during the development of colorectal cancer (CRC) and may play a crucial role in the process of field cancerization [3]–[5]. The theory of field cancerization describes the formation of a genetically and epigenetically altered, but histologically normal field around the primary tumor [6]–[8]. These genetic and epigenetic changes could contribute to the altered epithelial homeostasis, characterized by increased cell proliferation and predispose to the development of cancer in morphologically normal adjacent tumor (NAT) areas [9]. In some cases, between tumoral and NAT areas, a transitional area (TA) was identified, which displayed a different degree of dysplasia (i.e. altered crypt morphology, elongation, pseudostratification, loss of cell polarity and nuclear polymorphism). Although several studies have already described molecular abnormalities in association with field cancerization in epithelial tumors including CRC, the exact role of stroma in this process is still unclear [3], [10]. Here, we aim to examine the potential role of stroma-derived Wnt inhibitor secreted frizzled-related protein 1 (*SFRP1*) in CRC field cancerization.

SFRP1 inhibits proliferation and induces apoptosis by directly binding to Wnt-1 and Wnt-5 ligands via preventing the activation of Wnt receptors (i.e. Frizzled receptors (FRZ) and low-density lipoprotein receptor-related protein-5 and 6 (LRP-5, LRP-6)) [11]. This system is dysregulated in around 90% of sporadic CRC patients due to aberrant canonical Wnt signaling, including mutation of cytoplasmic β-catenin degradation complex (DC) proteins, such as Adenomatous Polyposis Coli (APC) and Axin [12]–[14]. In 5% of CRC cases, β-catenin is mutated and does not undergo proteasomal degradation via failed phosphorylation by GSK3β (member of DC) [15]. Mutation of the Wnt pathway results in inappropriate nuclear β-catenin migration, accumulation and T-cell factor (TCF)/lymphocyte enhanced factor (LEF) activation [11], [13]. This irregular TCF/LEF activation is independent of Wnt receptor activation; however, changes in the homeostasis of cell lines bearing an APC mutation as a result of the effect of different Wnt inhibitors have been described [12], [16], [17]. Based on the methylation analysis of macrodissected samples, it has been described that in colorectal carcinogenesis *SFRP1* promoter is epigenetically silenced [12], [18]–[20].

In this study, we aim to examine the protein expression and methylation patterns of myofibroblast-derived *SFRP1* in NAT and CRC tissues, and to demonstrate the effect of SFRP1 protein on HCT116 CRC cell line as a potential model of paracrine (stromal) inhibition of the Wnt pathway in colorectal carcinoma.

## Materials and Methods

### Ethics statement

The study was conducted according to the declaration of Helsinki and approved by Semmelweis University Ethics Committee and the governmental Regional and Institutional Committee of Science and Research Ethics (TUKEB), Nr:69/2008). Written informed consent was obtained from all patients included in the study.

### mRNA microarray analysis of biopsy and laser microdissected stroma samples

Endoscopically obtained biopsy samples from CRC (stage II, moderately differentiated tumors from sigmoid colon and rectum; n = 49) areas and paired histologically normal colonic mucosa (n = 49) were taken during routine colonoscopy and stored in RNALater Reagent (Qiagen Inc, Germantown, US) at −80°C until further processing. Total RNA was extracted and Affymetrix microarray analysis was performed as described before [21]. In the laser capture microdissection study, surgically removed NAT (n = 6) and CRC (n = 6) samples were used, which were embedded in TissueTek OCT compound (Sakura Finetek, Japan). Series of 6 µm sections were mounted onto PALM Membrane Slide 1.0 PEN (Carl Zeiss, Bernried, Germany) at −20°C and were stored at −80°C. Slides were fixated in 70% and absolute ethanol, then stained with cresyl violet acetate (Sigma-Aldrich, St. Louis, USA). Cells were collected from the stromal section in 5 biological replicates using the PALM Microbeam system (PALM, Bernried, Germany). The microarray experiment was performed as previously described [22]. All .cel files are available at GEO (Gene Expression Omnibus, http://www.ncbi.nlm.nih.gov/geo/) under access number: GSE4183 and GSE37364.

### Cell culture, proliferation and apoptosis assays

Human CRC cell line HCT116 (from István Peták M.D., Ph.D., 1st Department of Pathology and Experimental Cancer Research, Semmelweis University, Budapest, Hungary) were cultured in DMEM (Sigma-Aldrich, Irvine, UK) supplemented with L-glutamine, 10% fetal bovine serum (FBS, Sigma-Aldrich, Irvine, UK) and 1% penicillin-streptomycin mixture (Sigma-Aldrich, Irvine, UK), then grown at 37°C in an atmosphere of 5% CO_2_ and 95% humidity.

For the assays, cells were seeded at a density of 70,000 cells/cm^2^ in collagen-treated 24-well culture plates (Greiner Bio One, Frickenhausen, Germany) and treatments were carried out in duplicate (using three wells for each concentration). Cells were treated for 48 hours with SFRP1 full length recombinant protein (Abcam, ab64445, Cambridge, UK) at concentrations of 0.1 µg/ml and 1.0 µg/ml. During the treatment fetal bovine serum deprived cultures were used to avoid the interaction with SFRP1.

The treated and control cells were harvested and fixed for 30 minutes at room temperature in 70% ethanol (−20°C) and stored at −20 °C until further analysis. DNA was extracted with alkaline buffer (200 mM di-sodium phosphate, pH 7.8, adjusted with 200 mM citric acid) supplemented with 100 µg/ml RNase A (Sigma-Aldrich, Irvine, UK) followed by 10 µg/ml propidium iodide (Sigma-Aldrich, Irvine, UK) staining and incubated for 15 minutes at room temperature. 10,000–20,000 events were measured per sample by FACScan flow cytometer (Becton Dickinson FACScan, CA, USA), and the analyses were performed by Winlist software (Verity Software House).

### Immunohistochemistry

For immunohistochemistry normal biopsy samples (n = 20), surgically removed CRC (n = 35) and colonic tissues containing NAT and CRC areas (n = 14) were used. Samples were fixed in formaldehyde, embedded in paraffin and 4 µm thick sections were cut. Following deparaffinization and rehydration, microwave-based antigen retrieval was performed in TRIS EDTA buffer (pH 9.0) (900 W/10 minutes, then 340 W/40 minutes). SFRP1 protein expression was detected using anti-SFRP1 polyclonal antibody (ab4193, Abcam, Cambridge, UK, 1∶500 dilution) with diaminobenzidine - hydrogen peroxidase - chromogen-substrate system (Cytomation Liquid DAB + Substrate Chromogen System, K3468, Dako, CA, USA). This antibody showed high specificity against SFRP1 protein which was confirmed by Western blot (http://www.abcam.com/sfrp1-antibody-ab4193.html). Scoring of SFRP1 protein expression representing the intensity of the immunohistochemical reaction (both in myofibroblasts and epithelial cells) was made on the basis of the following criteria: the scoring value was −2 for no reaction, 0 for weak, 1 for moderate, and 2 for strong cytoplasmic protein expression.

To identify the stromal source of SFRP1 protein α-SMA/SFRP1 dual fluorescent staining was used in samples that contained NAT tissues and CRC areas as well (n = 9). The slides were incubated with anti-α-SMA (Dako, CA, USA ) 1∶1 solution with Alexa Fluor 488 (Invitrogen, Eugene, CA, USA) and anti-SFRP1 (ab4193, Abcam, Cambridge, UK) 1∶500 dilution with Alexa Fluor 546; A11035, Invitrogen, Eugene, USA) polyclonal antibodies. Slides were digitally archived using a Pannoramic Scan instrument (software version 1.11.25.0, 3DHISTECH Ltd, Budapest, Hungary), and analyzed with a digital microscope software (Pannoramic Viewer, v. 1.11.43.0 3DHISTECH Ltd, Budapest, Hungary). We counted 1000 α-SMA+ stromal cells in representative regions of samples with the Marker Counter built-in modem. Where TA was identifiable, the counted cell number was lower (150–300) because of the limited size of these areas. One-way ANOVA test was used for data analysis.

### DNA methylation analysis of laser microdissected α-SMA positive myofibroblasts and epithelial cells

Surgically removed stage II, moderately differentiated CRC (n = 8) and paired NAT (n = 8) samples (3 slides/sample) were snap-frozen in liquid nitrogen and stored at −80°C until further use. 1,000 myofibroblasts/slides (6 samples; 18 slides) were collected based on their α-SMA positivity, while epithelial cells were readily recognizable without specific staining (H&E stain; 10 samples; 30 slides). To analyze the methylation status of the *SFRP1* gene promoter, laser capture microdissected samples were bisulfite converted using the EZ DNA Methylation-Direct Kit^™^ (Zymo Research, Irvine, USA) according to the manufacturer's protocol. A region on the promoter was amplified using unbiased BS-PCR primers that do not differentiate between methylated and unmethylated bisulfite modified DNA. (F: 5′-GGAAAGAGATAAGGGGAGAAAAAGAA-3′; R: 5′-ATTTCATAAATTTACAAATATAATCCAAACTCC-3′). These primers were designed using PyroMark Assay Design software 2.0. They amplify a region 230 bp upstream of the transcription startpoint that contains 6 CpG sites (Figure S1). The specificity of the primers was tested *in silico* by the BiSearch software.

PCR cycling and HRM were performed on a LightCycler 480 instrument (Roche, Basel, Switzerland). The 25 µl reaction mix consisted of a final concentration of 1x AmpliTaq Gold 360 PCR Master Mix (Life Technologies, Carlsbad, USA), 1x ResoLight HRM Dye (Roche, Basel, Switzerland), 200 nM forward and reverse primers and 1 ng/well bisulfite converted DNA template. Cycling conditions were as follows: 95°C (10 min), 95°C (30 s), 60°C (30 s) decreased by 0,5°C in every cycle, 72°C (30 s) for 10 touchdown PCR cycles followed by, 95°C (30 s), 56°C (30 s) and 72°C (30 s) in 40 PCR cycles. On completion of the PCR thermal cycling, for the HRM analysis the samples were denatured at 95°C for 1 min, cooled down to 40°C and held for 1 min, then continuously warmed up to 95°C at a rate of 0.03°C/second during the melting curve fluorescence acquisition. For calibration of the analysis, 0% and a 100% artificially methylated control DNA samples were used (EpiTect PCR Control DNA Set, Qiagen, Hilden, Germany). To identify characteristic melting profiles of DNA products the LightCycler 480 Instrument Tm Calling analysis software Module was used.

## Results

### Reduced stromal *SFRP1* mRNA expression in CRC compared to NAT

**Figure 1:**
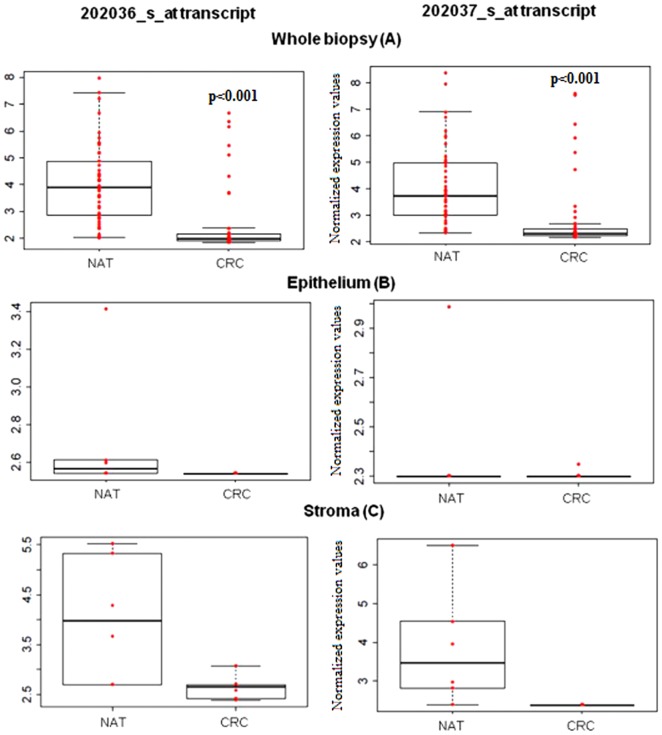
Gene expression of *SFRP1* in epithelium and stroma of NAT and CRC samples (202036_s_at and 2020367_s_at transcripts). According to normalized log2 fluorescence intensities, biopsy samples from NAT showed high *SFRP1* expression, which was significantly (P<0.001) reduced in CRC samples (**A**). In epithelial cells only weak SFRP1 expression was detected both in NAT and CRC (**B**). In stroma *SFRP1* expression was significantly increased (**C**). In this area a different scale was applied for data visualization.

Whole biopsy (macrodissected) samples from NAT and CRC regions were compared. Macrodissected NAT samples showed significantly (P<0.001) higher *SFRP1* mRNA expression than CRC samples (Figure 1/A). Using laser microdissected tissue compartments, higher (although not statistically significant) *SFRP1* mRNA expressions were found in the NAT epithelium compared to CRC epithelium (Figure 1/B). However, a significant (P<0.001) difference was observed in the expression of *SFRP1* transcripts (202036_s_at and 202037_s_at) between stromal samples from NAT and CRC. The average gene expression values in CRC was one-third (0.38 and 0.36 respectively) of the average expression values observed in NAT samples (Figure 1/C, Table 1). Abnormal Wnt signaling in parallel with reduced *SFRP1* was analyzed with Affymetrix whole genome microarrays. Based on 38 normal and 38 CRC samples (GSE37364), genes with significantly different (logFC >|2|, p<0.001) expression levels in the tumors with reduced SFRP1 expression compared to healthy controls were listed in Table S1.

**Table 1:** Summary of DNA methylation, mRNA and protein expression changes of *SFRP1* gene and protein in the stromal compartment.

	analyzed cells	NAT	Tumor	P
**mRNA expression**	stroma (LCM)	logFC_CRC-N_ = −2.23		<0.001
		n = 38	n = 38	
**DNA methylation**	α-SMA myofibroblasts (LCM)	0%	60%	n.a.
		n = 3/3	n = 3/3	
**protein expression**	α-SMA+ SFRP1+/α-SMA cells (LCM)	85.37%±12.6%	27.65%±18.2%	<0.001
		n = 9/9	n = 9/9	

LCM: laser capture microdissection.

### Stromal and epithelial SFRP1 protein expression in normal, NAT, TA and CRC

**Figure 2:**
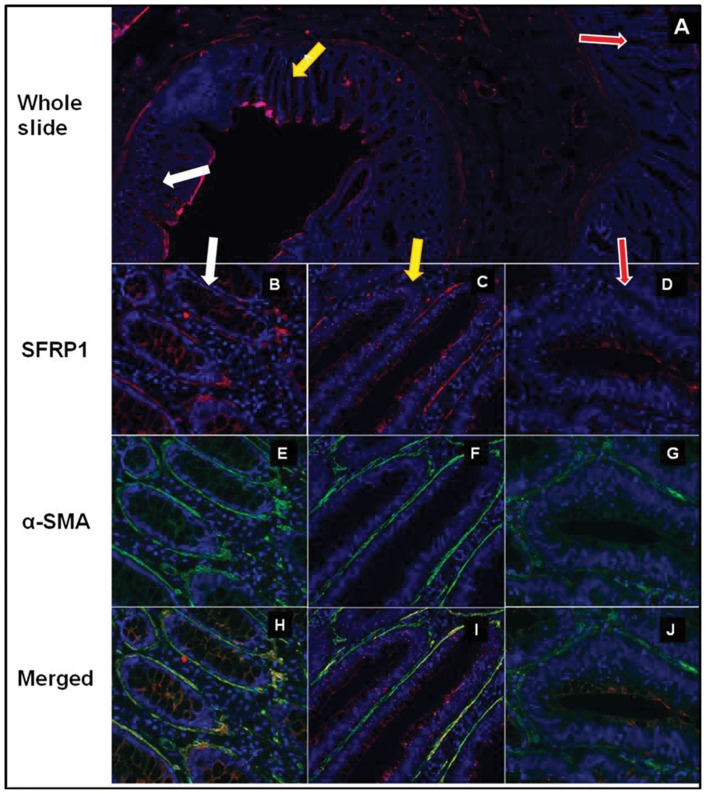
SFRP1 protein expression in α-SMA+ myofibroblasts in NAT, TA and CRC areas in representative photomicrographs. SFRP1 protein expression (red immunofluorescent staining) in whole slide (**A**) which includes NAT (white arrow), TA (yellow arrow) and CRC (red arrow). Both in NAT region (in 9/9 cases; **B**) and TA (in 4/9 cases; **C**) α-SMA+ myofibroblasts (**E, F**) showed retained SFRP1 expression (85.73±12.61%) (**B, C**; merged pictures: **H, I**). Around most CRC glands α-SMA+ myofibroblasts (green immunofluorescent staining; **G**) showed reduced (P<0.001) SFRP1 expression (27.65%±18.27%; in 9/9 cases) (**D**; merged picture: **J**).

Strong stromal SFRP1 protein expression (score +2) was found in normal, NAT and TA tissues, which localized mainly in the pericryptal region. These stromal SFRP1+ cells were identified as α-SMA+ myofibroblasts in NAT and in TA by dual fluorescent immunohistochemistry. Both α-SMA and SFRP1 showed strong (score +2) or moderate (score +1), diffuse, cytoplasmic staining in the elongated stromal cells. In NAT regions (9/9 cases) pericryptal α-SMA+ cells showed strong SFRP1 protein expression (85.73±12.61%; all α-SMA and SFRP1 double positive cells/all α-SMA positive cells in stroma; Figure 2/B, E, H, Table 1). Interestingly pericryptal α-SMA+ myofibroblasts retained strong or moderate SFRP1 protein expression in TAs (this area was identifiable in 4/9 cases), but the frequency of α-SMA/SFRP1 double positve cells was lower (72.32±2.32%) than in NAT tissues (Figure 2/C, F, I). In most of the analyzed CRC samples heterogeneous SFRP1 expression was detected. The number of α-SMA+ cells increased significantly, but the SFRP1 protein expression was significantly reduced (27.65%±18.27%) in representative tumoral areas (in 9/9 cases) as compared to NAT (Figure 2/D, G, J, Table 1).

**Figure 3:**
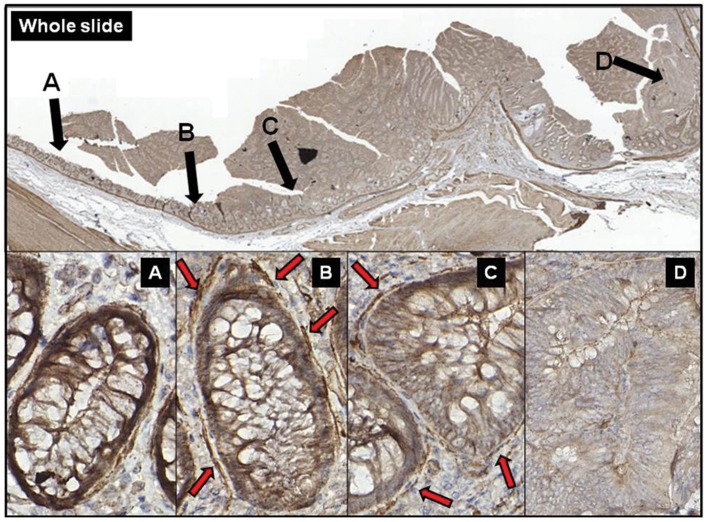
Change of epithelial SFRP1 protein expression with relative distance to the tumor in representative photomicrographs. The expression of SFRP1 protein is almost equivalent in the epithelial layer and in the subepithelial myofibroblasts in normal areas (**A**). In NAT areas which were localized near the tumor (**B**) and TA (**C**) the epithelium showed decreased SFRP1 protein expression, while the subepithelial myofibroblasts retained their SFRP1 protein expression (**red arrows**). In high-grade dysplasia both the epithelial and the myofibroblast-derived SFRP1 protein expression significantly decreased (**D**).

In normal samples epithelial SFRP1 expression was moderate (score: +1), lower than it was found in myofibroblasts (score: +2). Decreased SFRP1expression was observed in 57.14% of NAT samples (8 of 14 samples) in crypts which localized closely to the tumor (Figure 3/B) and TAs (Figure 3/C). Parallel with the disappearance of epithelial SFRP1 protein expression, subepithelial myofibroblasts showed retained protein expression in these areas (Figure 3/B, C). NAT regions which localized more distantly from CRC showed similar SFRP1 protein expression patterns than normal samples (Figure 3/A). Decreased epithelial SFRP1 protein expression (score: 0) was detected in 71.42% of CRC samples (34 of 14+35 samples).

### SFRP1 recombinant protein induces apoptosis in HCT116 cells

We analyzed the apoptotic effect of SFRP1 recombinant protein in HCT116 (APC wild type, β-catenin mutant) cells by flow cytometry. As the anti-proliferative and apoptotic effect of SFRP1 is well-known [12], [23], we examined these functions of SFRP1 at low concentrations (exogenously administered recombinant human SFRP1 (rhSFRP1; Abcam, Cambridge, UK); 0.1 µg/ml and 1.0 µg/ml) as a potential model of paracrine signaling. Comparative analysis of treated versus non-treated specimens showed measurable (both not statistically significant) increase of apoptosis in a dose-dependent manner (control: 10.66±0.7; 0.1 µg/ml: 12.29±0.88; 1.0 µg/ml: 16.13±1.68) as measured 48 hours after a single dose of rhSFRP1 protein (Figure S2).

### SFRP1 promoter hypermethylation in the NAT area and CRC

**Figure 4:**
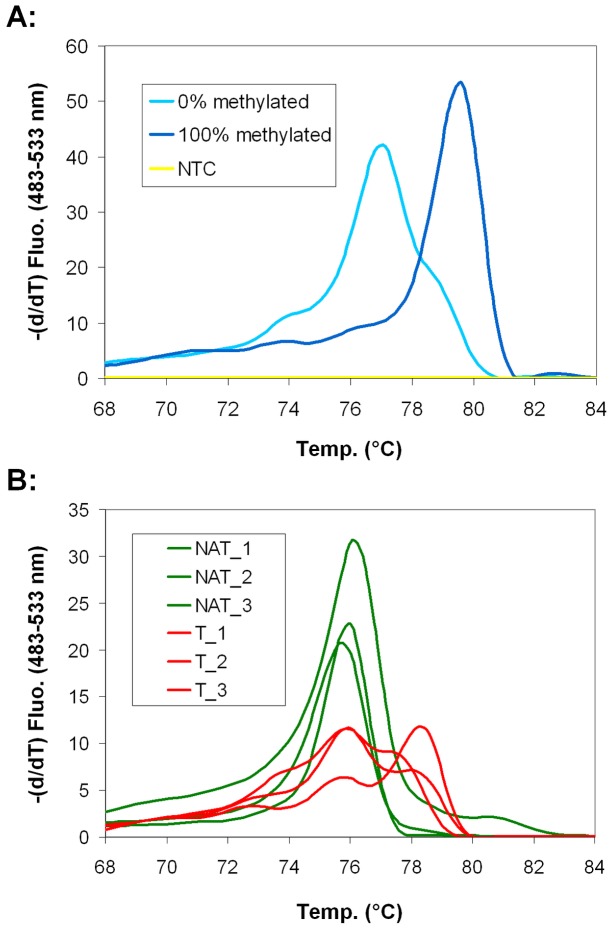
Promoter hypermethylation of *SFRP1* gene in BS-PCR HRM. *SFRP1* is hypermethylated in α-SMA positive myofibroblasts in CRC. **A:** Melting peaks of *SFRP1* BS-PCR products. Melting temperature (Tm) of 0% methylated standard sample was 77.1°C, while Tm of 100% methylated standard was 79.6°C. **B:** There was only one unmethylated melting peak detectable for NAT samples (green curves). In CRC samples (red curves) an additional melting peak belonging to hypermethylated products appeared, suggesting that α-SMA positive myofibroblasts are heterogenous concerning *SFRP1* promoter methylation.

The methylation status of α-SMA immunopositive stromal myofibroblasts was examined in laser microdissected cells from NAT and CRC samples. The BS-PCR products of 100% methylated control with a higher melting temperature (melting peak: 79.6°C) and unmethylated control with a lower melting temperature (melt peak: 77.1°C) (Figure 4/A) were used. Myofibroblasts from NAT showed only one melting peak in the unmethylated temperature range, while myofibroblasts from CRC had an additional peak at higher temperature indicating a partially hypermethylated status of *SFRP1* promoter in CRC (Figure 4/B, Table 1). Double peaks on the melting temperature (Tm) curves of CRC samples denoted their heterogeneity considering their methylation status, which was probably due to the presence of cells with unmethylated promoters among hypermethylated promoters. Decreased epithelial SFRP1 protein expressions were found in crypts of most NAT samples which localized closely to the tumor. This decreased protein expression was related to epigenetic silencing of *SFRP1* gene. Partially *SFRP1* promoter hypermethylation was found in morphologically normal gland epithelium in 40% (2 of 5 samples) of NAT samples (Figure S3).

## Discussion

Wnt signaling is a major regulator of a variety of cellular processes during embryonic development and promotes tissue homeostasis in the adult. Wnts are secreted lipid-modified glycoproteins regulating a wide range of cellular behavior including differentiation, proliferation, migration, survival, polarity and stem cell self-renewal [24]. Altered Wnt signaling may contribute to the development of several disorders including cancer [25]. The canonical/β-catenin pathway is the most extensively studied Wnt signaling mechanism, which is triggered by Wnt binding to a member of the Frizzled receptor family and co-receptors (LRP5 or LRP6) [26]. This results in the recruitment of Dishevelled to Frizzled and Axin to phosphorylated LRP5/6, leading to the dissociation of a β-catenin degradation complex. In the absence of Wnt this complex mediates the sequential phosphorylation of β-catenin, causing its ubiquitination and proteasomal degradation. Wnt stimulation allows the accumulation of hypophosphorylated β-catenin in the cytosol and its translocation into the nucleus, where it binds to TCF/LEF and promotes the expression of Wnt/β-catenin target genes [26]. Constitutive activation of this pathway is commonly present in many types of cancer [24], [25]. Non-canonical Wnt-signaling pathways (planar cell polarity or increased intracellular calcium concentration) are transduced by Frizzleds and/or other Wnt receptors or co-receptors [26], [27]. Several non-canonical Wnt signaling mechanisms have been reported to inhibit the β-catenin pathway by decreasing β-catenin/TCF association with DNA or increasing β-catenin turnover [28]–[30].

SFRPs comprise a family of five proteins in mammals that were first identified as antagonists of the Wnt/β-catenin pathway during embryonic development [31]–[33]. SFRPs possess a remarkable range of biological activities, including tumor suppression [11], [34]. This is also strengthened by epigenetic silencing of SFRP gene expression in a wide variety of cancers [35], [36], and supported by the observation that restoration of expression is suppressive of the tumor phenotype [37], [38]. By contrast, SFRP overexpression has been observed in some of the same malignancies [39]–[41]. Consistent with this duality, SFRP1 showed a biphasic effect on β-catenin stabilization elicited by Wingless, increasing β-catenin protein levels at low SFRP1 concentrations, but inhibiting it at high concentrations [42]. In different cellular contexts, SFRP1 has been shown either to increase or decrease β-catenin stabilization [43], [44]. Furthermore, another study suggested that SFRP1 could stimulate the Wnt/calcium pathway via Frizzled-2 independently of endogenous Wnts [45].

Regarding field cancerization, methylation of *hMLH1*, *CDKN2A/P16* and *SFRP1* has been recently indicated to be associated with malignant transition in endometrial cancer [46].

In our study, the apoptotic effect of SFRP1 protein was demonstrated by administering low doses of rhSFRP1 on HCT116 CRC cell line. In HCT116 cells rhSFRP1 protein caused a measurable increase in apoptosis.

We investigated the role of SFRP1 regarding the stroma-epithelium interaction in CRC and NAT areas. SFRP1 protein is a well-known intercellular inhibitor of Wnt pathway which regulates the epithelial proliferation as autocrine and/or paracrine signal [12], [23]. We identified α-SMA+ myofibroblasts as the main source of stromal SFRP1 protein in NAT regions, in parallel with unmethylated *SFRP1* promoter sequences. We also observed moderate to strong SFRP1 protein expression in α-SMA+ myofibroblasts in TA, which was characterized by altered crypt morphology (e.g. elongation, pseudostratification, loss of cell polarity and nuclear polymorphism). On the contrary, epigenetic silencing of epithelial *SFRP1* appeared in both areas and this could serve as an early sign of field cancerization.

According to our hypothesis, in these areas field cancerization caused epithelial proliferation, which was partly inhibited by the less dysregulated stroma, indicated by the lower frequency of *SFRP1* hypermethylation in myofibroblasts. Thus, decreased epithelial SFRP1 protein expression may partly be replaced by stromal sources (i.e. by α-SMA+ myofibroblasts). In contrast to the DNA repair gene O6-methylguanine-DNA methyltransferase (*MGMT*) [10], which has been found to be methylated in the macroscopic field of some CRCs, in our study epithelial *SFRP1* hypermethylation was only present in areas closely localized to the tumor (NAT).

Although in most CRC cases stromal myofibroblasts exhibited hypermethylation and low protein expression of *SFRP1*, we hypothesize that the remaining myofibroblasts with intact SFRP1 protein expression can decrease the proliferation rate of Wnt mutant CRC cells.

To conclude, our present study indicates that SFRP1 protein expression is conserved in α-SMA+ myofibroblasts and partially disappears in the epithelium of NAT and TA. In contrast, we found low SFRP1 immunopositivity stroma-associated myofibroblasts in CRC. Both epithelial and myofibroblast-derived *SFRP1* expression is under epigenetic regulation. We presume that the paracrine, anti-proliferative effect of myofibroblast-derived SFRP1 may inhibit field cancerization in NAT and TA areas, and so cancer-associated myofibroblasts may support the uncontrolled tumor cell proliferation by an epigenetically silenced Wnt inhibitory signal.

## Supporting Information

Figure S1 **Position of the BS-PCR product (black) on the SFRP1 gene promoter.** The transcript of SFRP1 is represented by the blue line; CpG sites are red lines. Figure modified from UCSC genome browser (Human, GRCh37/hg19 Assembly).
(TIF)

Figure S2 **Apoptotic effect of recombinant SFRP1 protein on HCT116 cell line.** rhSFRP1 protein caused measurable (both not statistically significant) increase in apoptosis (48 hours after a single dose).
(TIF)

Figure S3 **Promoter hypermethylation of** ***SFRP1*** **in epithelial cells in NAT area.** A: Melting peaks of *SFRP1* BS-PCR products. Melting temperature of the 0% methylated standard sample was 77°C, while Tm of the 100% methylated standard was 79.5°C. B: *SFRP1* was hypermethylated in 2/5 epithelial cells (40%) in NAT area (red line) indicated by their melting peaks at higher temperature.
(TIF)

Table S1 Genes with significantly different (logFC >|2|, p<0.001) expression levels in tumors with reduced *SFRP1* expression compared to healthy controls.
(DOCX)

## References

[1] Shtilbans V (2013) Role of Stromal-Epithelial Interaction in the Formation and Development of Cancer Cells. Cancer Microenviron 6: 193–202. 23430817 10.1007/s12307-013-0131-5 PMC3855375

[2] Powell DW, Mifflin RC, Valentich JD, Crowe SE, Saada JI, et al (1999) Myofibroblasts. I. Paracrine cells important in health and disease. Am J Physiol 277: 1–9. 10.1152/ajpcell.1999.277.1.C1 10409103

[3] Ge L, Meng W, Zhou H, Bhowmick N (2010) Could stroma contribute to field cancerization? Med Hypotheses 75: 26–31. 20149555 10.1016/j.mehy.2010.01.019

[4] Powell DW, Mifflin RC, Valentich JD, Crowe SE, Saada JI, et al (1999) Myofibroblasts. II. Intestinal subepithelial myofibroblasts. Am J Physiol 277: 183–201. 10.1152/ajpcell.1999.277.2.C183 10444394

[5] Trujillo KA, Heaphy CM, Mai M, Vargas KM, Jones AC, et al (2011) Markers of fibrosis and epithelial to.mesenchymal transition demonstrate field cancerization in histologically normal tissue adjacent to breast tumors. Int J Cancer 129: 1310–1321. 21105047 10.1002/ijc.25788 PMC3249233

[6] Braakhuis BJ, Tabor MP, Kummer JA, Leemans CR, Brakenhoff RH (2003) A genetic explanation of Slaughter's concept of field cancerization: evidence and clinical implications. Cancer Res 63: 1727–1730. 12702551

[7] Ushijima T (2007) Epigenetic field for cancerization. J Biochem Mol Biol 40: 142–150. 17394762 10.5483/bmbrep.2007.40.2.142

[8] Patai AV, Molnár B, Kalmár A, Schöller A, Tóth K, et al (2012) Role of DNA methylation in colorectal carcinogenesis. Dig Dis 30: 310–315. 22722557 10.1159/000337004

[9] Bernstein C, Bernstein H, Payne CM, Dvorak K, Garewal H (2008) Field defects in progression to gastrointestinal tract cancers. Cancer Lett 260: 1–10. 18164807 10.1016/j.canlet.2007.11.027 PMC2744582

[10] Shen L, Kondo Y, Rosner GL, Xiao L, Hernandez NS, Vilaythong J, et al (2005) MGMT promoter methylation and field defect in sporadic colorectal cancer. J Natl Cancer Inst 97: 1330–1338. 16174854 10.1093/jnci/dji275

[11] Rubin JS, Barshishat-Kupper M, Feroze-Merzoug F, Xi ZF (2006) Secreted WNT antagonists as tumor suppressors: pro and con. Front Biosci 11: 2093–2105. 16720296 10.2741/1952

[12] Suzuki H, Watkins DN, Jair KW, Schuebel KE, Markowitz SD, et al (2004) Epigenetic inactivation of SFRP genes allows constitutive WNT signaling in colorectal cancer. Nat Genet 36: 417–422. 15034581 10.1038/ng1330

[13] Clevers H (2004) Wnt breakers in colon cancer. Cancer Cell 5: 5–6. 14749120 10.1016/s1535-6108(03)00339-8

[14] Schneikert J, Behrens J (2007) The canonical Wnt signalling pathway and its APC partner in colon cancer development. 56: 417–425. 10.1136/gut.2006.093310 PMC1856802 16840506

[15] Morin PJ, Sparks AB, Korinek V, Barker N, Clevers H, et al (1997) Activation of b-catenin-Tcf signaling in colon cancer by mutations in beta-catenin or APC. Science 275: 1787–1790. 9065402 10.1126/science.275.5307.1787

[16] He B, Reguart N, You L, He B, Reguart N, You L (2005) Blockade of Wnt-1 signaling induces apoptosis in human colorectal cancer cells containing downstream mutations. Oncogene 24: 3054–3058. 15735684 10.1038/sj.onc.1208511

[17] de Sousa EM, Vermeulen L, Richel D, Medema JP (2011) Targeting Wnt signaling in colon cancer stem cells. Clin Cancer Res 17: 647–653. 21159886 10.1158/1078-0432.CCR-10-1204

[18] Bovolenta P, Esteve P, Ruiz JM, Cisneros E, Lopez-Rios J (2008) Beyond Wnt inhibition: new functions of secreted Frizzled-related proteins in development and disease. J Cell Sci 121: 737–746. 18322270 10.1242/jcs.026096

[19] Qi J, Zhu YQ, Luo J, Tao WH (2006) Hypermethylation and expression regulation of secreted frizzled-related protein genes in colorectal tumor. World J Gastroenterol 12: 7113–7117. 17131472 10.3748/wjg.v12.i44.7113 PMC4087771

[20] Tanaka J, Watanabe T, Kanazawa T, Tada T, Kazama Y, et al (2008) Silencing of secreted frizzled-related protein genes in MSI colorectal carcinogenesis. Hepatogastroenterology 55: 1265–1268. 18795670

[21] Galamb O, Wichmann B, Sipos F, Spisák S, Krenács T, et al (2012) Dysplasia-carcinoma transition specific transcripts in colonic biopsy samples. PLoS One 7: e48547. 23155391 10.1371/journal.pone.0048547 PMC3498283

[22] Spisák S, Kalmár A, Galamb O, Wichmann B, Sipos F, et al (2012) Genome-wide screening of genes regulated by DNA methylation in colon cancer development. PLoS One 7: e46215. 23049694 10.1371/journal.pone.0046215 PMC3462205

[23] Hughes KR, Sablitzky F, Mahida YR (2011) Expression profiling of Wnt family of genes in normal and inflammatory bowel disease primary human intestinal myofibroblasts and normal human colonic crypt epithelial cells. Inflamm Bowel Dis 17: 213–220. 20848536 10.1002/ibd.21353

[24] Clevers H, Nusse R (2012) Wnt/β-catenin signaling and disease. Cell 149: 1192–1205. 22682243 10.1016/j.cell.2012.05.012

[25] Anastas JN, Moon RT (2013) WNT signalling pathways as therapeutic targets in cancer. Nat Rev Cancer 13: 11–26. 23258168 10.1038/nrc3419

[26] Niehrs C (2012) The complex world of WNT receptor signalling. Nat Rev Mol Cell Biol 13: 767–779. 23151663 10.1038/nrm3470

[27] Kikuchi A, Yamamoto H, Sato A, Matsumoto S (2011) New insights into the mechanism of Wnt signaling pathway activation. Int Rev Cell Mol Biol 291: 21–71. 22017973 10.1016/B978-0-12-386035-4.00002-1

[28] Topol L, Jiang X, Choi H, Garrett-Beal L, Carolan PJ, et al (2003) Wnt-5a inhibits the canonical Wnt pathway by promoting GSK-3-independent beta-catenin degradation. J Cell Biol 162: 899–908. 12952940 10.1083/jcb.200303158 PMC2172823

[29] Kühl M, Geis K, Sheldahl LC, Pukrop T, Moon RT, et al (2001) Antagonistic regulation of convergent extension movements in Xenopus by Wnt/beta-catenin and Wnt/Ca2+ signaling. Mech Dev 106: 61–76. 11472835 10.1016/s0925-4773(01)00416-6

[30] Ishitani T, Ninomiya-Tsuji J, Nagai S, Nishita M, Meneghini M, et al (1999) The TAK1-NLK-MAPK-related pathway antagonizes signalling between beta-catenin and transcription factor TCF. Nature 399: 798–802. 10391247 10.1038/21674

[31] Leyns L, Bouwmeester T, Kim SH, Piccolo S, De Robertis EM (1997) Frzb-1 is a secreted antagonist of Wnt signaling expressed in the Spemann organizer. Cell 88: 747–756. 9118218 10.1016/s0092-8674(00)81921-2 PMC3061830

[32] Wang S, Krinks M, Lin K, Luyten FP, Moos M Jr (1997) Frzb, a secreted protein expressed in the Spemann organizer, binds and inhibits Wnt-8. Cell 88: 757–766. 9118219 10.1016/s0092-8674(00)81922-4

[33] Bovolenta P, Esteve P, Ruiz JM, Cisneros E, Lopez-Rios J (2008) Beyond Wnt inhibition: new functions of secreted Frizzled-related proteins in development and disease. J Cell Sci 121: 737–746. 18322270 10.1242/jcs.026096

[34] Cowling VH, D'Cruz CM, Chodosh LA, Cole MD (2007) c-Myc transforms human mammary epithelial cells through repression of the Wnt inhibitors DKK1 and SFRP1. Mol Cell Biol 27: 5135–5146. 17485441 10.1128/MCB.02282-06 PMC1951955

[35] Suzuki H, Gabrielson E, Chen W (2002) A genomic screen for genes upregulated by demethylation and histone deacetylase inhibition in human colorectal cancer. Nat Genet 31: 141–149. 11992124 10.1038/ng892

[36] Dahl E, Wiesmann F, Woenckhaus M, Stoehr R, Wild PJ, Veeck J, et al (2007) Frequent loss of SFRP1 expression in multiple human solid tumours: association with aberrant promoter methylation in renal cell carcinoma. Oncogene 26: 5680–5691. 17353908 10.1038/sj.onc.1210345

[37] Gumz ML, Zou H, Kreinest PA, Childs AC, Belmonte LS, et al (2007) Secreted frizzled-related protein 1 loss contributes to tumor phenotype of clear cell renal cell carcinoma. Clin Cancer Res 13: 4740–4749. 17699851 10.1158/1078-0432.CCR-07-0143

[38] Zi X, Guo Y, Simoneau AR, Hope C, Xie J, Holcombe RF, et al (2005) Expression of Frzb/secreted Frizzled-related protein 3, a secreted Wnt antagonist, in human androgen-independent prostate cancer PC-3 cells suppresses tumor growth and cellular invasiveness. Cancer Res 65: 9762–9770. 16266997 10.1158/0008-5472.CAN-05-0103

[39] Lee JL, Chang CJ, Wu SY, Sargan DR, Lin CT (2004) Secreted frizzled-related protein 2 (SFRP2) is highly expressed in canine mammary gland tumors but not in normal mammary glands. Breast Cancer Res Treat 84: 139–149. 14999144 10.1023/B:BREA.0000018412.83348.ff

[40] Joesting MS, Perrin S, Elenbaas B, Fawell SE, Rubin JS, et al (2005) Identification of SFRP1 as a candidate mediator of stromal-to-epithelial signaling in prostate cancer. Cancer Res 65: 10423–10430. 16288033 10.1158/0008-5472.CAN-05-0824

[41] Saini S, Liu J, Yamamura S, Majid S, Kawakami K, et al (2009) Functional significance of secreted Frizzled-related protein 1 in metastatic renal cell carcinomas. Cancer Res 69: 6815–6822. 19723665 10.1158/0008-5472.CAN-09-1254

[42] Uren A, Reichsman F, Anest V, Taylor WG, Muraiso K, et al (2000) Secreted frizzled-related protein-1 binds directly to Wingless and is a biphasic modulator of Wnt signaling. J Biol Chem 275: 4374–4382. 10660608 10.1074/jbc.275.6.4374

[43] Wawrzak D, Métioui M, Willems E, Hendrickx M, de Genst E, et al (2007) Wnt3a binds to several sFRPs in the nanomolar range. Biochem Biophys Res Commun 357: 1119–1123. 17462603 10.1016/j.bbrc.2007.04.069

[44] Yokota T, Oritani K, Garrett KP, Kouro T, Nishida M, et al (2008) Soluble frizzled-related protein 1 is estrogen inducible in bone marrow stromal cells and suppresses the earliest events in lymphopoiesis. J Immunol 181: 6061–6072. 18941195 10.4049/jimmunol.181.9.6061 PMC2735054

[45] Rodriguez J, Esteve P, Weinl C, Ruiz JM, Fermin Y, et al (2005) SFRP1 regulates the growth of retinal ganglion cell axons through the Fz2 receptor. Nat Neurosci 8: 1301–1309. 16172602 10.1038/nn1547

[46] Di Domenico M, Santoro A, Ricciardi C Iaccarino M, Iaccarino S, et al (2011) Epigenetic fingerprint in endometrial carcinogenesis: the hypothesis of a uterine field cancerization. Cancer Biol Ther 12: 447–457. 21709441 10.4161/cbt.12.5.15963

