# Supplementary figures and images for: Myofibroblast-Derived SFRP1 as Potential Inhibitor of Colorectal Carcinoma Field Effect
Source: PLoS One. 2014 Nov 18;9(11):e106143. doi: 10.1371/journal.pone.0106143 (PMC4236006; doi:10.1371/journal.pone.0106143)

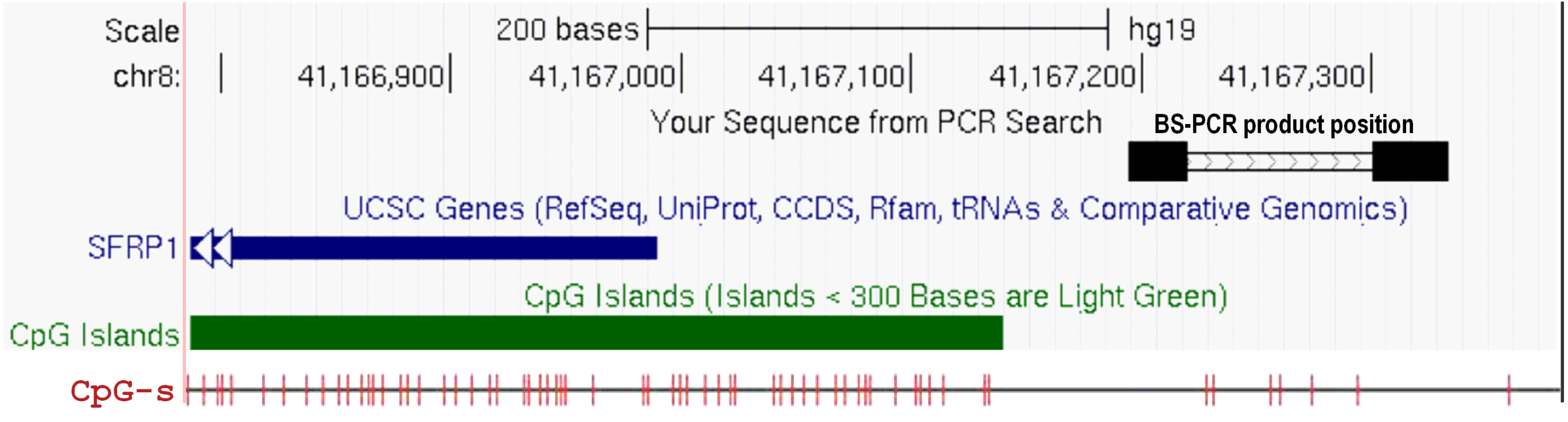

Supplement: Figure S1 — Position of the BS-PCR product (black) on the SFRP1 gene promoter. The transcript of SFRP1 is represented by the blue line; CpG sites are red lines. Figure modified from UCSC genome browser (Human, GRCh37/hg19 Assembly). (TIF) [file pone.0106143.s001.tif]

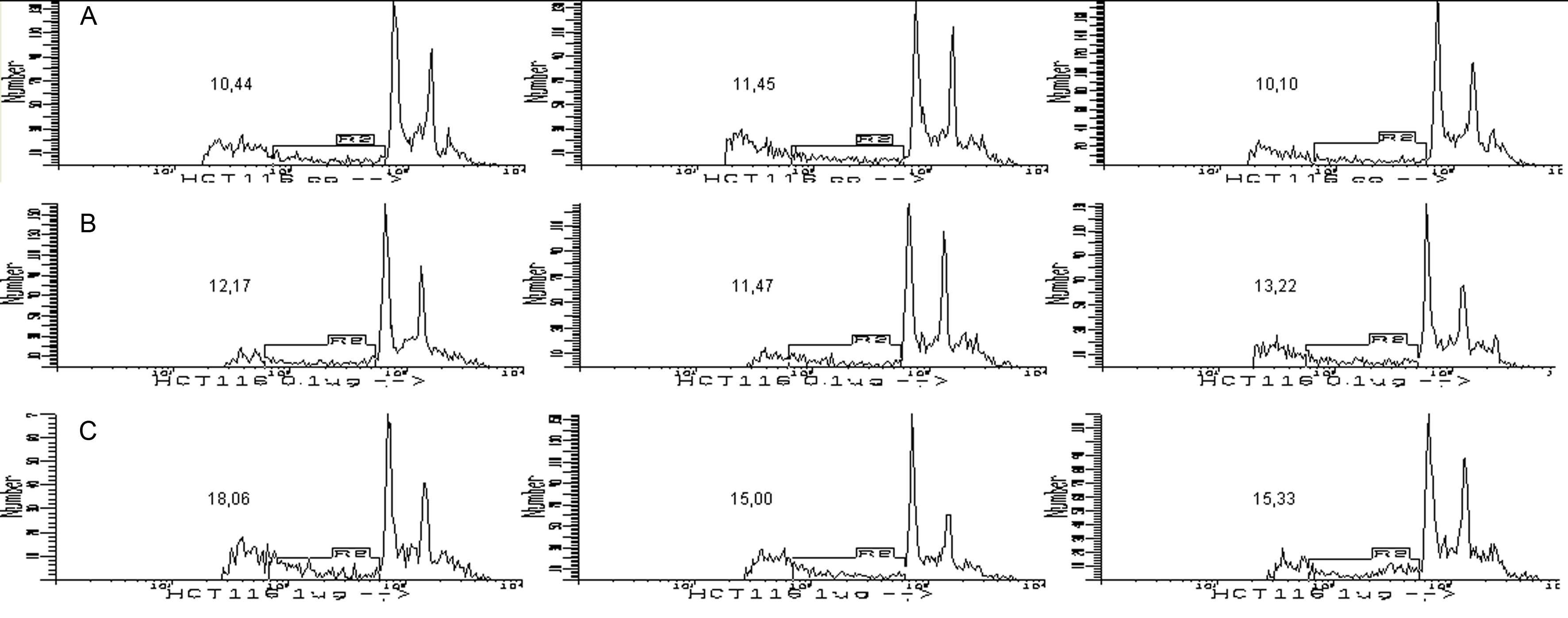

Supplement: Figure S2 — Apoptotic effect of recombinant SFRP1 protein on HCT116 cell line. rhSFRP1 protein caused measurable (both not statistically significant) increase in apoptosis (48 hours after a single dose). (TIF) [file pone.0106143.s002.tif]

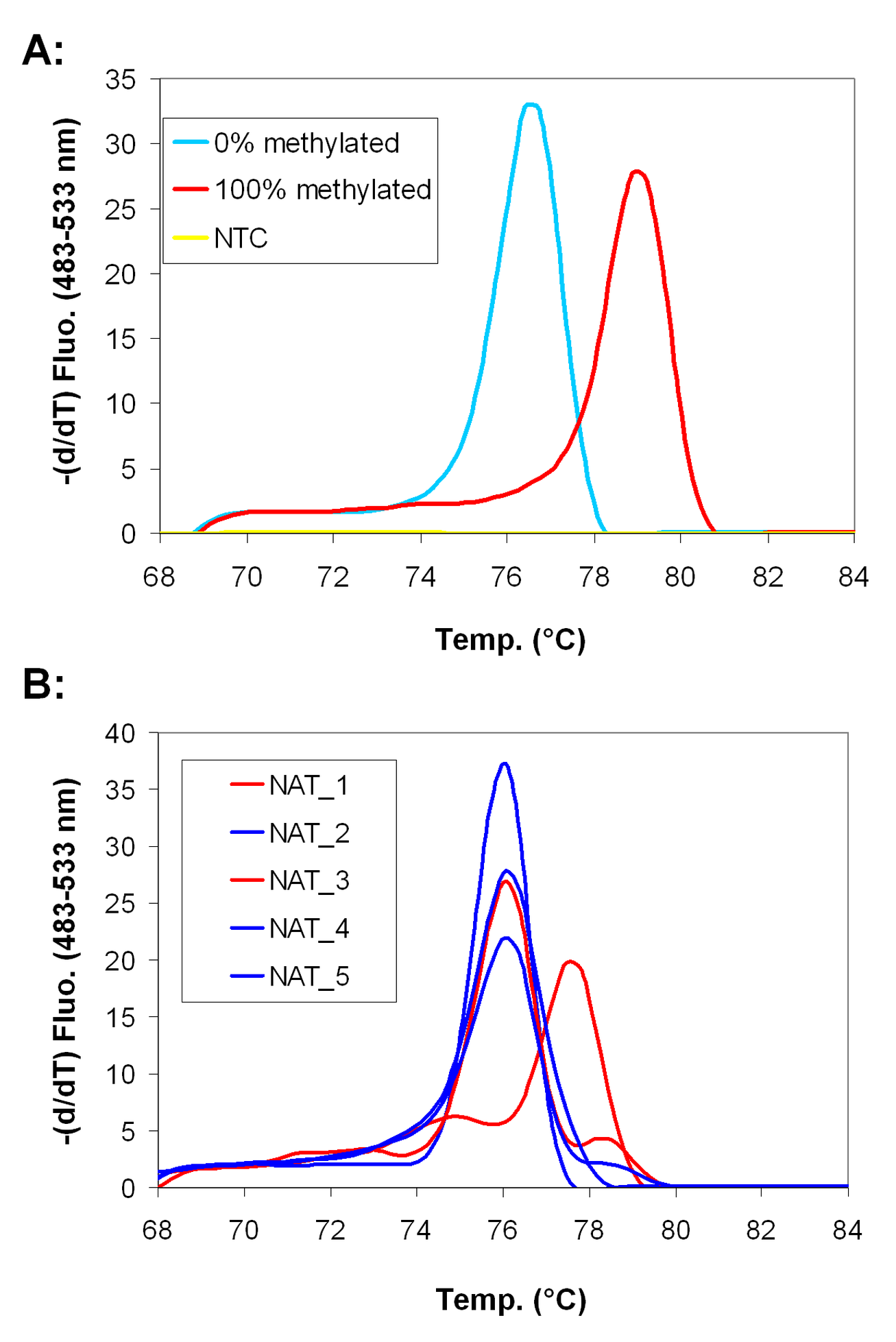

Supplement: Figure S3 — Promoter hypermethylation of SFRP1 in epithelial cells in NAT area. A: Melting peaks of SFRP1 BS-PCR products. Melting temperature of the 0% methylated standard sample was 77°C, while Tm of the 100% methylated standard was 79.5°C. B: SFRP1 was hypermethylated in 2/5 epithelial cells (40%) in NAT area (red line) indicated by their melting peaks at higher temperature. (TIF) [file pone.0106143.s003.tif]
