# Supplementary material for: Myofibroblast-Derived SFRP1 as Potential Inhibitor of Colorectal Carcinoma Field Effect
Source: PLoS One. 2014 Nov 18;9(11):e106143. doi: 10.1371/journal.pone.0106143 (PMC4236006; doi:10.1371/journal.pone.0106143)
Supplement: Table S1 — Genes with significantly different (logFC >|2|, p<0.001) expression levels in tumors with reduced SFRP1 expression compared to healthy controls. (DOCX) [file pone.0106143.s004.docx]

**Supplementary Table 1.** Genes with significantly different (logFC>│2│, p<0.001) expression levels in tumors with reduced *SFRP1* expression compared to healthy controls.

A: Overexpressed Wnt pathway genes in CRC as compared to normal controls.

B: Underexpressed Wnt pathway genes in CRC as compared to normal controls.
